# Interaction Between Intrinsic Renal Cells and Immune Cells in the Progression of Acute Kidney Injury

**DOI:** 10.3389/fmed.2022.954574

**Published:** 2022-07-07

**Authors:** Junhui Deng, Zhifen Wu, Yun He, Lirong Lin, Wei Tan, Jurong Yang

**Affiliations:** ^1^The Third Affiliated Hospital of Chongqing Medical University, Chongqing, China; ^2^The Fifth People's Hospital of Chongqing, Chongqing, China

**Keywords:** acute kidney injury, intrinsic renal cells, immune cells, interaction, microenvironment

## Abstract

A growing number of studies have confirmed that immune cells play various key roles in the pathophysiology of acute kidney injury (AKI) development. After the resident immune cells and intrinsic renal cells are damaged by ischemia and hypoxia, drugs and toxins, more immune cells will be recruited to infiltrate through the release of chemokines, while the intrinsic cells promote macrophage polarity conversion, and the immune cells will promote various programmed deaths, phenotypic conversion and cycle arrest of the intrinsic cells, ultimately leading to renal impairment and fibrosis. In the complex and dynamic immune microenvironment of AKI, the bidirectional interaction between immune cells and intrinsic renal cells affects the prognosis of the kidney and the progression of fibrosis, and determines the ultimate fate of the kidney.

## Introduction

Acute kidney injury (AKI) refers to a clinical syndrome in which renal function rapidly declines in a short period of time due to various pathological factors. During the pathophysiological process of AKI, all cells in the kidney, including renal cells, resident immune cells, and infiltrating immune cells, undergo various adaptive responses mediated by complex and unclear mechanisms (1). In recent studies, single-cell sequencing of kidneys from animal models of AKI revealed spatial co-localization of renal intrinsic cells with immune cells from all stages of disease (2–5). Various studies have shown that a bidirectional interaction between renal innate cells and immune cells exists, and plays a key role in the mechanisms governing renal injury and renal repair (6, 7). In this review, we will summarize the interaction between renal intrinsic cells and immune cells in the development of AKI and provide insights into its clinical significance.

## Intrinsic Cells Promote Immune Cell Infiltration

Under physiological conditions, a small number of innate immune cells reside in the normal human kidney, including macrophages, dendritic cells (DCs) and lymphocytes (8). Following injury to renal tubular epithelial cells (TECs) and endothelial cells, resident immune cells are activated and recruit circulating immune cells to infiltrate the kidneys (9–11). Renal tissue biopsies from AKI patients with sepsis were stained pathologically and displayed a large infiltration of neutrophils and macrophages within the glomeruli and tubulointerstitial regions of the kidney (12). These findings were corroborated by another study where single-cell RNA sequencing of kidney tissues from lipopolysaccharide (LPS)-induced mouse models of AKI also detected large numbers of macrophages, neutrophils, and T lymphocytes in diseased animals (13, 14). Furthermore, neutrophils, macrophages, dendritic cells, NK cells, T cells, and B cells have been reported to be recruited and activated in mouse and rat models of ischemia-reperfusion AKI (15, 16), and in cisplatin-induced AKI, with early mast cell infiltration also noted (17).

Renal intrinsic cells release various cytokines and chemokines, such as C-C motif chemokine ligand 2 (CCL2), CCL20, and C-C motif chemokine receptor 5 (CCR5) upon cell death, and release danger-associated molecular patterns (DAMPs) and activating pattern recognition receptors (PRRs), which in turn promote infiltration of renal tissue by a large number of immune cells (18–24). It has been reported that in renal injury due to ischemia/reperfusion (I/R), necrotic tubular epithelial cells and endothelial cells release DAMPs such as histones, heat shock proteins, high mobility histone (HMGB1), hyaluronic acid and biglycan, and secrete chemokines after direct interaction with Toll-like receptors 2 (TLR2) and TLR4, further driving neutrophil recruitment to the kidney (25–31). Infiltrating neutrophils interact with activated platelets to form neutrophil extracellular traps (NETs) that contribute to renal tissue injury (32). Peptidyl arginine deiminase-4 (PAD4) is a member of the peptidyl arginine deiminase family, whose function is to convert arginine in proteins to citrulline, and is an important regulator of the formation of NETs (33, 34). Rabadi et al. have repeatedly reported that in addition to being expressed in immune cells, ischemia-reperfusion induces high expression of PAD4 in TECs. This promotes nuclear translocation of PAD4 to the cytoplasm through interaction with NF-κB, and promotes neutrophil infiltration and NET formation (35, 36).

NF-κB is a key regulator of innate and adaptive immunity, and an important signaling factor for renal innate cells to promote immune cell infiltration. Highlighting its role in the inflammatory response during AKI, TEC specific NF-κB knockout mice had attenuated neutrophil and macrophage infiltration in I/R-injured AKI kidneys compared to wild-type control mice, and decreased expression vascular endothelial growth factor A(VEGFA), C-X-C motif chemokine ligand 1(CXCL1), and CXCL2 in cultured NF-κB knockout TECs *in vitro* (37). Moreover, Yu et al. found that NF-κB also regulated the expression of RANTES (a member of the CC chemokine family) in I/R-injured mouse renal tubular cells, promoting the release of inflammatory factors tumor necrosis factor α (TNF-α), lnterleukin-1β (IL-1β), and monocyte chemotactic protein 1 (MCP-1), thereby promoting the infiltration of T lymphocytes and macrophages into the renal interstitium (38). In addition to NF-κB, transforming growth factor β (TGF-β) signaling in TECs could also promote immune cell infiltration. By constructing mice expressing TGF-β specific receptor 1 in renal tubular epithelial cells, the researchers detected macrophages, dendritic cells, monocytes and T cells infiltration and tubular damage after stimulating the activation of TGF-β signaling pathway, confirming that renal TGF-β signaling in TECs is sufficient to induce renal tissue inflammation and lead to AKI (39).

Renal intrinsic cells can also promote infiltration by macrophages, neutrophils, and other monocytes throughIL-1.IL-33, an IL-1 family member involved in the pathogenesis of AKI, is released by endothelial cells in the kidneys of I/R-injured and cisplatin-induced AKI mice. It acts by directly targeting invariant natural killer T (iNKT) cells via binding to its receptor ST2 on the cell surface, and induces interferon (IFN) and IL-17A production, which promotes neutrophil infiltration and activation (40, 41). IL-34 is also produced and released by TECs after I/R injury in the kidney, promoting the proliferation of intrarenal and bone marrow derived macrophages, and the infiltration of circulating monocytes into the kidney through the release of chemokines, TNF-α, and MCP-1 (42). The expression and release of various cytokines and chemokines CCL2, TNF-α, and CXCL10 in TECs are regulated by the transcription factor GATA Binding Protein 2 (GATA2), a zinc finger-containing transcription factor that is specifically expressed in renal tubular cells, and maintain its cellular identity. In the study by Yu et al. (43) CCL2, TNF-α, and CXCL10 were downregulated in GATA2-deficient mice, and monocyte infiltration was significantly reduced in renal tissue. In addition to the secretion of cytokines and chemokines, TECs also communicate with immune cells via exosomes. Following injury to renal tubular epithelial cells, CD26-containing exosomes are produced and bind to the chemokine receptor C-X-C motif chemokine receptor 4 (CXCR4), leading to upregulation of downstream chemokine pathways, increased stromal-derived factor 1 (SDF1), and macrophage and neutrophil infiltration (44). TECs can also amplify the inflammatory response through the release of exosomes that contain chemokine CCL2 mRNA, which are directly transferred to macrophages, leading to enhanced macrophage migration (45).

## Intrinsic Renal Cells Promote Macrophage Polarization

Recent studies have shown that the phenotype and function of macrophages are highly plastic and can play a key role in the process of AKI and repair by altering their phenotype (46–48). Macrophages are divided into M1 (classical activation type) and M2 (selective activation type) according to their activation status. M1 macrophages differentiate in response to interferon γ (IFNγ) and/or LPS. They express high levels of nitric oxide synthase (iNOS) and produce pro-inflammatory cytokines such as TNF-α, IL-16, and IL-12 that contribute to M1 macrophage antibacterial and tumor-killing functions. In contrast, M2 macrophages are induced by IL-4/IL-13 and express high levels of insulin-like growth factor 1 (IGF-1), mannose receptor 1 (CD206), and arginase 1 (ARG-1). M2 macrophages are anti-inflammatory and have wound healing functions (49–53).

Early in AKI, TECs subjected to various injuries exert pro-inflammatory effects mainly through the release of exosomal miRNAs that transmit signals and regulate the induction of M1 macrophages. Hypoxic injury stimulates the expression of hypoxia-inducible factor-1α (HIF-1α) in renal tubular epithelial cells and promotes the release of miRNA-23a-rich exosomes to induce M1 macrophage differentiation by inhibiting the ubiquitin editor A20 (54). Interestingly, inhibition of miRNA-23a attenuated tubulointerstitial inflammation before I/R injury in mice, and lead to reduced exosome-mediated transport of miRNA-23a between TECs and macrophages.LvLL et al. found that TECs-derived exosomal miRNA-19b-3p also promoted the activation of M1 macrophages during renal injury. Specifically, TEC-derived exosomal miR-19b-3p internalized by macrophages increased NF-κB/suppressor of cytokine signaling (1SOCS-1) activation, and induced tubulointerstitial inflammation in mice that could be reversed by mIR-19b-3p inhibition (55). Clinically, high levels of miR-19b-3p positively correlated with the severity of tubulointerstitial inflammation, further confirming that exosomal miR-19b-3p mediates communication between damaged TECs and macrophages (55).

During later stages of AKI, IL-4-stimulated arginase 1(ARG-1) and CD206-positive M2 macrophages predominate and promote the transition from tubular injury to tubular repair. *In vitro* studies have demonstrated that renal tubular cells co-cultured with IL-4-stimulated M2 macrophages, but not IFNγ-stimulated pro-inflammatory M1 macrophages, induce tubular cell proliferation (56). Colony stimulating factors (CSF) are a group of growth factors that promote the proliferation and differentiation of myeloid progenitor cells, including macrophage colony stimulating factor (M-CSF/CSF1), granulocyte-macrophage colony stimulating factor (GM-CSF/CSF2), and granulocyte colony stimulating factor (G-CSF/CSF3) (57). In mouse models of AKI induced by I/R injury or novel diphtheria toxin (DT), CSF1 secreted by renal proximal tubular epithelial cells stimulated enhanced polarization of M2 macrophages, and mediated regenerative repair of renal tubular epithelium after AKI (58). Wang et al. demonstrated that proximal tubular CSF-1-specific knockout mice had reduced M2 macrophage polarization, delayed renal function and structural recovery, and increased tubular interstitial fibrosis in the kidney after I/R injury (59). In another study, CSF2 expression was significantly increased in HK-2 and human M1 macrophage co-cultures (60). CSF2 is secreted by HK-2 cells, but not by M1 macrophages. However, culturing macrophages with exogenous recombinant CSF2 promotes the transition from an M1 to M2 phenotype by activating phosphorylated signal transducer and activator of transcription 5(p-STAT5) in a dose- and time-dependent manner. Intraperitoneal injection of CSF2-neutralizing antibodies exacerbate renal injury and inhibit tubular proliferation, thereby decreasing survival. In contrast, administration of recombinant mouse CSF2 protein dampens septic AKI. Endogenous erythropoietin (EPO), which is synthesized and secreted by the peritubular fibroblasts, is thought to be an important protective protein in AKI that protects renal tissue by reducing apoptosis of TECs and decreasing inflammation (61, 62). It has been reported that in rhabdomyolysis-associated AKI, EPO promotes macrophage polarization toward the M2 phenotype via the Janus Kinase 2(JAK2)/STAT3/STAT6 pathway in response to IL-4 stimulation, thereby reducing M1 damage to the relevant tissues and enhancing the healing and repair mechanisms of M2 macrophages (63).

## Immune Cells Promote Various Types Of Programmed Cell Deaths Of Renal Intrinsic Cells

TECs undergo various types of programmed cell death (PCD) including apoptosis, pyroptosis, necroptosis, and ferroptosis (64–67). Upon sensing local tissue injury, resident and infiltrating inflammatory cells in the kidney promote TEC PCD by producing and releasing large amounts of inflammatory cytokines and activating various pathway proteins, leading to TEC detachment, tubular occlusion, and tubular inactivation. In the early stage of AKI, M1 macrophages secrete various pro-inflammatory cytokines, including TNF-α and interleukins that binds to the corresponding receptors in TECs to promote PCD (68, 69). TNF-α has been reported to bind to the TNF receptor 1 on the surface of TECs to induce RIP3-dependent necrotizing apoptosis (70, 71). In I/R-induced AKI, TNFα has also been reported to exacerbate kidney injury through the receptor-interacting protein 3(RIP1)/RIP3 necroptosis signaling pathway (72). Type I interferon α (IFN-α) is a multifunctional active cytokine that induces apoptosis in renal TECs through activation of caspase-3, caspase-8, and caspase-9, leading to DNA fragmentation and nuclear condensation (73). Increased expression of IFN-α can be observed in renal biopsies of AKI patients after renal transplantation in clinical practice (74). Plasmacytoid DCs are a distinct DC subpopulation specialized in IFN production. Plasmacytoid DCs producing IFN-α directly induce apoptosis in TECs *in vitro*, suggesting that pDCs play a deleterious role in AKI via IFN-α (74). TLR9 in DCs can also be activated by mitochondrial DNA (mtDNA), by mediating IL-17A production in γδ T cells, thus promoting the development of AKI during sepsis (75). Furthermore, IL-17A has been shown to induce TEC apoptosis in AKI in sepsis. Interestingly, IL-17A knockout mice administered a cecum ligation puncture had reduced numbers of TUNEL-positive TECs, decreased cleaved caspase-3 levels, and an increased Bax/Bcl-2 expression ratio, suggesting that IL-17A plays a pathogenic role in AKI by inducing apoptosis in TECs (76). Macrophages can also regulate TEC pyroptosis by releasing exosomal miRNA. Chen et al. isolated exosomes from M1 and M2 macrophages, and miRNA sequencing revealed differential miRNA expression levels in M1 and M2 exosomes, in which miR-93-5p was involved in the regulation of mouse renal tubular epithelial cells pyroptosis. It was determined that thioredoxin-interacting protein (TXNIP) was a direct target of miR-93-5p in mouse renal tubular epithelial cells (77).

Mitogen-activated protein kinase (MAPK), an extracellular signal-regulated kinase, is another important pathway protein for macrophage function (78, 79). It is suggested that the MAPK signaling pathway plays a key role in kidney injury molecular 1(KIM-1) mediated renal injury by promoting macrophage phenotypic transformation and migration (80). Moreover, in macrophages cultured *in vitro*, LPS can induce inflammatory responses through activation of the MAPK pathway (81). Su et al. found that Pannexin 1 (PANX1), an ATP-releasing pathway family protein, mediates iron death in TECs via the MAPK pathway in I/R-induced AKI. Therefore, macrophages may regulate iron death in TECs by regulating MAPK (82).

## Immune Cells Promote Phenotypic Conversion Of Intrinsic Renal Cells

Epithelial mesenchymal transition (EMT) of renal tubular cells is one of the most important features of tubulointerstitial fibrosis during the transition from AKI to chronic kidney disease (CKD). Increased production of pro-fibrotic cytokines such as MCP-1, complement protein 3 (C3), and TGF-β after AKI promotes EMT and leads to renal fibrosis and eventual progression to CKD (83). The inflammatory microenvironment is critical for the progression of EMT in TECs. Protein and mRNA expression of α-smooth muscle actin (α-SMA) and waveform protein increased in HK-2 cells after co-culture with M2 macrophages in Transwell chambers for 48 h, while E-cadherin significantly decreased, confirming the promotional effect of M2 macrophages on EMT (84). The investigators found that cisplatin induced only incomplete EMT in TECs alone, while conditioned medium of cisplatin-treated fibroblasts and macrophages induced complete EMT in TECs. High expression levels of α-SMA and collagen-1 in cisplatin-activated fibroblasts were detected in the triple co-culture cell model, and increased expression of ARG-1 and CD206 by macrophages were observed, indicating M2 macrophage polarization and the development of complete EMT in TECs. These data reveal a synergistic effect of TECs, fibroblasts, and M2 macrophages to promote the progression of renal fibrosis (85). Furthermore, in a mouse unilateral ureteral obstruction (UUO) model, macrophages released high levels of TGF-β1 after polarization to M2 phenotypes that promoted EMT-induced renal fibrosis. In contrast, depletion of M2 macrophages specifically inhibited EMT and subsequently reduced renal fibrosis (86, 87).

During the transition from AKI to CKD, macrophages regulate tubule cell EMT through various mechanisms. The typical epithelial cell morphology of HK-2 cells disappears after co-culture with monocytes, accompanied by decreased E-cadherin expression and increased expression of α-SMA and fibronectin, suggesting that EMT occurs in HK-2 cells by a specific mechanism of monocyte upregulation through the NF-kB signaling pathway. This phenotype was related to the upregulation of intercellular adhesion molecule 1 (ICAM-1) expression in HK-2 cells and activation of the NF-kB signaling pathway (88). HMGB-1 is a chromatin-binding protein that acts as a signal transducer by regulating IL-1 and TNF expression under inflammatory conditions (89). Human proximal tubular epithelial cells cultured using conditioned medium containing supernatants from activated peripheral blood mono-nuclear cells showed significant upregulation of HMGB-1 and induction of EMT via TGF-β, including decreased E-calmodulin expression, increased α-SMA expression, and enhanced cell migration, suggesting that HMGB-1 is a key mediator of EMT in TECs.

The complement system is another important pathogenic mediator in the pathogenesis of AKI, and its member C3 regulates renal fibrosis through the HMGB1/TGF-β1/Smad2/3 signaling pathway. *In vitro* experiments revealed that C3 secreted by macrophages stimulated HMGB1 translocation from the nucleus to the cytoplasm and promoted the expression of TGF-β1 in TECs and induced epithelial cell EMT (90). A study by Zheng et al. demonstrated that matrix metalloproteinase (MMP) family members secreted by macrophages in the kidney are involved in the development and progression of renal fibrosis through EMT of TECs as well as activation of resident fibroblasts, endothelial-mesenchymal transition, and pericyte-myofibroblast trans-differentiation (91). Murine renal tubular epithelial cell lines and primary renal tubular epithelial cells cultured with conditioned medium from LPS-activated macrophages significantly increased MMP-9 expression. In contrast, blockade of MMP-9 resulted in EMT inhibition in TECs (92). These results were confirmed in mouse where MMP-9 was upregulated in the early and late stages of UUO in mice, with MMP-9 cleaved bone bridge protein and macrophage infiltration significantly reduced after MMP-9 blockade. Strikingly, MMP-9 blockade significantly reduced EMT of TECs and renal significantly improved renal fibrosis (93). Pentraxin3 (PTX3), an acute-phase protein produced by resident and innate immune cells, promotes renal fibrosis by upregulating TECs EMT via a c-JunN-terminal kinase (c-JNK)-dependent mechanism. Treatment of UUO in rats and HK-2 cells with recombinant human PTX3 (Rh-PTX3) resulted in time-dependent increases in PTX3, p-JNK and waveform protein levels, as well as decreases in E-cadherin, JNK phosphorylation, and EMT markers (94). CircRNA can also act as an important mediator of macrophage promotion of tubular cell EMT, as found in the UUO-induced mouse and *in vitro* HK-2 cell fibrosis models. CircACTR2 in macrophages promotes macrophage inflammation and induces EMT and fibrosis in TECs via miR-561 and NLRP3 inflammasome activation (95).

## Immune Cells Inhibit Tecs Cycle Arrest

The cell cycle encompasses the process of cell division and includes the G0, G1, S, G2, and M phases (96, 97). During AKI, TECs undergo cell cycle arrest in the G2/M phase and fail to differentiate, producing and secreting various pro-fibrotic factors such as TGF-β1 and connective tissue growth factor (CTGF). These factors act on peripheral cells and fibroblasts through the paracrine pathway, leading to poor renal repair and renal fibrosis (98–101). Regarding the role of immune cells and the cell cycle, it has been shown that M2 macrophages act on damaged TECs by releasing the Wnt ligand, Wnt7b, to bypass cell cycle arrest, promote cell proliferation, repair tubular basement membrane, and inhibit renal fibrosis (102).

Macrophage migration inhibitory factor (MIF), a pleiotropic cytokine in tissue injury, is produced and released by macrophages in AKI, and has been suggested to be an endogenous protective factor in the kidney (103). On MIF knockdown, TECs undergo G2/M cell cycle, expression of pro-inflammatory and pro-fibrotic mediators is increased, and renal fibrosis is exacerbated in mice with sepsis and UUO (104). It was recently found that the lipid transport protein 2 (Lcn-2) secreted by macrophages also plays a key role in TEC repair after AKI. In a co-culture system of NRK52E cells and bone marrow-derived macrophages, Lcn-2 inhibited TEC cell cycle arrest by inhibiting peroxisome proliferator-activated receptor-γ (PPAR-γ) and enhancing epithelial markers, ultimately promoting epithelial proliferation (105). Sirtuin 6 (SIRT6) is a NAD-dependent deacetylase that is highly unstable in macrophages, and can be rapidly degraded by the proteasome (106). Several studies have reported that SIRT6 expression is downregulated in multiple models of AKI, and exerts antioxidant effects and attenuates renal injury by regulating mitochondrial dynamics and inhibiting G2/M cell cycle arrest in TECs (107, 108). Trigger receptor in myeloid cells 1(TREM-1) is a regulator of TLR signaling and is highly upregulated in renal inflammatory macrophages (109). After knockdown of TREM-1 in I/R-injured renal tubular epithelial cells, the G2/M cell cycle is blocked leading to stress-induced senescence by a mechanisms associated with mitochondrial dysfunction and a decrease in cellular metabolic activity (110).

## Summary

**Figure 1:**
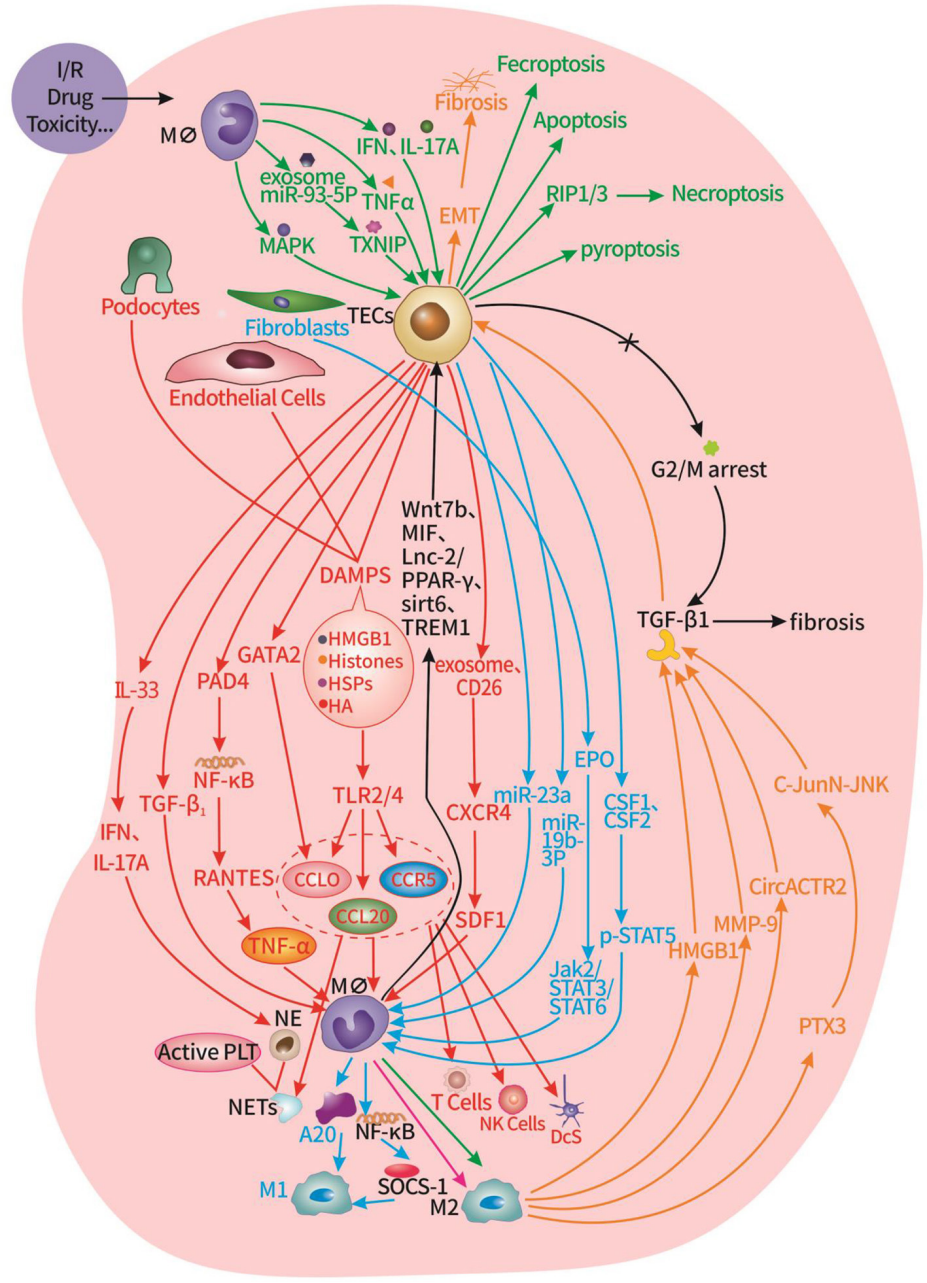
Interaction of innate cells and immune cells in the renal microenvironment of AKI. Red part: TECs, podocytes, endothelial cells promote the infiltration of macrophages, neutrophils, T cells, NK cells, DCs, and the formation of NETs. Blue part: TECs, fibroblasts promote the transformation of macrophages into M2 type. Green part: macrophages promote TECs apoptosis, pyroptosis, necroptosis, and ferroptosis. Orange part: macrophages promote TECs EMT. Black part: macrophages inhibit G2/M cycle arrest of TECs.

When the kidney is subjected to injury, renal intrinsic cells and inflammatory cells in the tissues promote and restrain each other through various mechanisms, presenting an intricate and dynamic microenvironmental state that ultimately determines cell fate decisions in the kidney (Figure 1). On the one hand, TECs, podocytes, endothelial cells, and fibroblasts promote the infiltration of immune cells in the circulation, and the polarization of macrophages, which amplifies the renal inflammatory response and promotes fibrosis after AKI. On the other hand, DCs and macrophages promote various programmed death of TECs, leading to renal tubular inactivation. Meanwhile, macrophages also balance the progression of renal fibrosis by promoting EMT and inhibiting G2/M cycle arrest of TECs.The current understanding of the interactions between renal intrinsic cells and inflammatory cells in pathology of AKI is only the tip of the iceberg, and deserves to be explored more extensively by researchers. Understanding the mechanisms mediating renal and immune cell interactions will provide new ideas and more effective targets for the prevention, intervention, and treatment of AKI.

## Author Contributions

JD and JY wrote the first draft. ZW, YH, LL, and WT reviewed and finalized the content of the manuscript. All authors read and approved the final version.

## Funding

This research was funded by the National Natural Science Foundation of China (No. 81770682) and the Chongqing Talent Program Project (cstc2021ycjh-bgzxm0090).

## Conflict of Interest

The authors declare that the research was conducted in the absence of any commercial or financial relationships that could be construed as a potential conflict of interest.

## Publisher's Note

All claims expressed in this article are solely those of the authors and do not necessarily represent those of their affiliated organizations, or those of the publisher, the editors and the reviewers. Any product that may be evaluated in this article, or claim that may be made by its manufacturer, is not guaranteed or endorsed by the publisher.

## Abbreviations

AKI: acute kidney injury
CKD: chronic kidney disease
CXCL1: C-X-C Motif Chemokine Ligand 1
RIP3: Receptor-Interacting Protein 3
ARG-1: arginase 1
C3: complement protein 3
CCL2: Ligand 2
CCL20: C-C Motif Chemokine Ligand 20
CCR5: C-C Motif Chemokine Receptor 5
c-JNK: c-JunN-terminal kinase
CSF: Colony stimulating factors
CTGF: connective tissue growth factor
CXCL2: C-X-C Motif Chemokine Ligand 2
CXCR4: C-X-C Motif Chemokine Receptor 4
DAMPs: danger-associated molecular patterns
DCs: dendritic cells
DT: diphtheria toxin
EMT: Epithelial mesenchymal transition
EPO: Endogenous erythropoietin
GATA2: GATA Binding Protein 2
G-CSF: granulocyte colony stimulating factor
GM-CSF: granulocyte-macrophage colony stimulating factor
HIF-1α: hypoxia-inducible factor-1α
HMGB1: high mobility histone
I/R: ischemia/reperfusion
ICAM-1: intercellular adhesion molecule 1
IFN: interferon
IGF-1: insulin-like growth factor 1
IL-1: Interleukin-1
IL-33: Interleukin-33
IL-34: Interleukin-34
iNKT cell: invariant natural killer T cell
JAK2: Janus Kinase 2
KIM-1: Kidney injury molecular 1
Lcn-2: the lipid transport protein 2
LPS: ipopolysaccharide
MAPK: Mitogen-activated protein kinase
MCP-1: monocyte chemotactic protein 1
MIF: Macrophage migration inhibitory factor
MMP: matrix metalloproteinase
NETs: neutrophil extracellular traps
PAD4: Peptidyl arginine deiminase-4
PANX1: Pannexin 1
PCD: programmed cell death
PPAR-γ: peroxisome proliferator-activated receptor-γ
PRRs: pattern recognition receptors
p-STAT-5: phosphorylated signal transducer and activator of transcription 5
PTX3: Pentraxin3
SDF1: stromal-derived factor 1
SIRT6: Sirtuin 6
SOCS-1: Suppressor Of Cytokine Signaling 1
TECs: tubular epithelial cells
TGF-β: Transforming Growth Factor β
TLR: Toll-like receptors
TNF-α: Tumor Necrosis Factor α
TREM-1: Trigger receptor in myeloid cells 1
TXNIP: thioredoxin-interacting protein
Type I interferon α: IFN-α
UUO: unilateral ureteral obstruction
VEGFA: Vascular Endothelial Growth Factor A
α-SMA: α-smooth muscle actin.
